# Development and Immunogenicity Evaluation of Baculovirus-Expressed Feline Bocavirus VP2 Virus-like Particles Vaccine in a Mouse Model

**DOI:** 10.3390/microorganisms14091893

**Published:** 2026-08-26

**Authors:** Jia-You Xing, Zi-Xuan Fu, Wen-Jie Xu, Jing-Yang Li, Zi-Ji Wang, Yu-Xin Xiang, Jiang Wang, Sheng-Li Ming, Yue-Ting Zheng, Jian-Li Li, Lei Zeng

**Affiliations:** 1 College of Veterinary Medicine, Henan Agricultural University, Zhengzhou 450046, China; xingjiayou123@163.com (J.-Y.X.); sufang555552024@163.com (Z.-X.F.); x15736736983@163.com (W.-J.X.); 15515555783@163.com (J.-Y.L.); 13782255923@163.com (Z.-J.W.); 15890038343@163.com (Y.-X.X.); wangjiang@henau.edu.cn (J.W.); mingshengli@henau.edu.cn (S.-L.M.); yuetingzheng@126.com (Y.-T.Z.); 2 Key Laboratory of Animal Biochemistry and Nutrition, Ministry of Agriculture and Rural Affairs, Zhengzhou 450046, China; 3 Key Laboratory of Veterinary Biotechnology of Henan Province, Henan Agricultural University, Zhengzhou 450046, China

**Keywords:** feline bocavirus, baculovirus expression system, virus-like particle vaccines, immunogenicity, adjuvant, neutralizing antibody, BALB/c mice

## Abstract

Feline bocavirus (FBoV) is an emerging enteric virus associated with gastrointestinal diseases in cats and has attracted increasing attention in feline health. However, no commercial vaccine is currently available for the prevention and control of FBoV infection. VP2 is the major capsid protein of FBoV and represents a promising target for vaccine development. In this study, the FBoV VP2 protein was expressed using the insect baculovirus expression system. The purified VP2 protein self-assembled into virus-like particles (VLPs), which were formulated with Alum, ISA 206, or GEL 02 adjuvants to prepare VP2 VLP vaccines. The immunogenicity, cellular immune responses, antigen uptake, biodistribution, germinal center responses, and safety of the vaccines were evaluated in BALB/c mice. The results showed that all VP2 VLP vaccine formulations induced VP2-specific IgG antibodies and neutralizing antibodies, promoted B- and T-lymphocyte activation, enhanced dendritic cell maturation, and stimulated germinal center-related immune responses. Among the tested formulations, VP2+GEL 02 induced the strongest immune responses and showed favorable safety in mice. These findings demonstrate that FBoV VP2 exhibits favorable immunogenicity and represents a promising vaccine antigen for further development against feline bocavirus.

## 1. Introduction

Cats are among the most common companion animals, living in close contact with humans and playing an important role in providing emotional support. To date, the global population of domestic cats is estimated to exceed 600 million [1]. As important members of many households, feline health has received increasing attention [2]. Viral diarrhea is common in cats [3], particularly in kittens, and represents a major threat to feline health. Although feline parvovirus (FPV) is considered a major viral cause of diarrhea in cats [4], other enteric viral infections are frequently detected in diarrheic cats [5,6,7], including FBoV [6,8,9]. FBoV belongs to the family *Parvoviridae*, the subfamily *Parvovirinae*, and the genus *Bocavirus* [10]. The feline bocaparvovirus species includes three genotypes, namely FBoV-1, FBoV-2, and FBoV-3, with FBoV-1 being more commonly detected. In 2012, FBoV was first detected in a stray cat in Hong Kong [8]. Subsequently, in 2015, FBoV was reported in Northeast China, where it was detected in fecal samples from cats with severe enteritis [11]. To date, FBoV has been reported in multiple countries, including China [5,11], Japan [12], Portugal [2], and the United States [13], and has been detected in fecal samples from both symptomatic and asymptomatic cats. Diarrhea is the most commonly reported clinical manifestation associated with FBoV infection [5]. FBoV infection has also been linked to severe pathological lesions, including hemorrhagic enteritis and systemic lymphoid tissue necrosis [6]. More recently, increasing evidence has also suggested a potential association between FBoV infection and neurological disorders, indicating that the virus may exhibit neurotropic properties [14]. Coinfection with FBoV-1 and FPV is frequently observed and may contribute to more severe clinical outcomes, such as hemorrhagic enteritis [6,11,15]. FBoV is an emerging pathogen in cats that has been associated with gastrointestinal and respiratory diseases. Owing to its increasing detection in domestic cats and its potential impact on feline health, the development of effective preventive strategies against FBoV warrants further investigation.

FBoV is a linear, single-stranded, non-enveloped DNA virus with an icosahedral morphology and a diameter of approximately 25–26 nm [16]. Its genome is approximately 5.5 kb in length and contains three open reading frames (ORFs): ORF1 encodes the non-structural protein NS1, ORF2 encodes the capsid proteins VP1 and VP2, and ORF3 encodes the nuclear phosphoprotein NP1, similar to other parvoviruses [17]. As the major structural protein of FBoV, VP2 plays a critical role in determining viral antigenicity, host adaptation, and pathogenicity. Structural variations in VP2 may contribute to antigenic drift and changes in bocavirus virulence [18]. Therefore, VP2 is the target protein for the development of vaccines.

Vaccination remains one of the most effective strategies for preventing and controlling infectious diseases. However, there is currently no commercial vaccine against FBoV. Efficient production of recombinant proteins is essential for the development of subunit vaccines. Baculoviruses are double-stranded DNA insect viruses that can efficiently express exogenous genes and produce complex eukaryotic proteins [19]. Owing to its versatility, safety, and cost-effectiveness, the baculovirus expression vector system (BEVS) has been widely used as a rapid and adaptable platform for the production of heterologous proteins, thereby facilitating vaccine development and the prevention of pathogen infections. VLPs are self-assembled nanostructures composed of viral structural proteins that closely resemble native virions but lack viral genetic material, making them highly safe and immunogenic vaccine platforms. Owing to their ability to efficiently induce both humoral and cellular immune responses, VLP-based vaccines have been successfully developed for several veterinary pathogens, including porcine circovirus type 2 (PCV2), foot-and-mouth disease virus (FMDV), and feline calicivirus (FCV). These successful applications demonstrate the considerable potential of the VLP platform for veterinary vaccine development. Therefore, the development of an FBoV VP2 VLP vaccine represents a promising strategy for preventing FBoV infection.

In addition, adjuvants are essential components of vaccines, as they can enhance immunogenicity and modulate the type of immune response. In this study, we used the classical aluminum adjuvant, together with two adjuvants commonly used in veterinary practice, ISA 206 and GEL 02. The Al(OH)_3_ adjuvant is an insoluble gel suspension commonly referred to as an aluminum adjuvant or alum adjuvant. As one of the most widely used vaccine adjuvants, Al(OH)_3_ has a long history of application in various vaccines and is known to induce strong humoral immune responses. Montanide™ ISA 206 is a water-in-oil-in-water (W/O/W) emulsion adjuvant with good tolerability and flowability [20]. Montanide™ GEL 02 is a ready-to-dilute polymeric adjuvant designed for aqueous vaccines [21]. These adjuvants have been reported to enhance antigen-specific immune responses and are widely used in the development of veterinary vaccines.

In this study, a safe and efficient baculovirus expression system was used as a protein expression platform to express the FBoV VP2 protein, which can self-assemble into VLPs. Subsequently, FBoV VP2 VLPs were formulated with the Alum, Montanide™ ISA 206, or Montanide™ GEL 02 adjuvant to prepare VLP vaccines, and their immunogenicity was evaluated in BALB/c mice. The results showed that the FBoV VP2 VLP vaccines induced effective humoral and cellular immune responses and elicited high levels of neutralizing antibodies. Given the absence of a commercially available vaccine against FBoV, the development of a safe and effective vaccine is urgently needed. Therefore, the objective of this study was to develop a baculovirus-expressed FBoV VP2 virus-like particle (VLP) vaccine and to evaluate its immunogenicity and safety in BALB/c mice.

Based on these considerations, we hypothesized that baculovirus-expressed FBoV VP2 VLPs could elicit robust humoral and cellular immune responses, particularly when formulated with appropriate adjuvants. Therefore, the objectives of this study were to develop an FBoV VP2 VLP vaccine using the baculovirus expression system and to systematically evaluate its immunogenicity, safety, antigen persistence, and immune responses when induced by different adjuvants in a BALB/c mouse model.

## 2. Materials and Methods

### 2.1. Cells, Virus and Antibodies

Sf9 cells (Cellosaurus, CVCL_0549) were cultured in a serum-free SIM SF medium (Sino Biological Inc., Beijing, China) at 27 °C and used for amplification of the recombinant baculovirus. High Five (Hi5; Cellosaurus, CVCL_C190) insect cells were cultured in a SIM HF medium (Sino Biological Inc., Beijing, China) at 27 °C with shaking at 135 rpm and used for protein expression. CRFK cells were maintained in Eagle’s minimum essential medium (EMEM; BNCC360906, BNCC, Beijing, China), whereas DC2.4 cells (Cellosaurus, CVCL_J409) were maintained in Dulbecco’s modified Eagle’s medium (DMEM; 11995, Solarbio, Beijing, China). Both media were supplemented with 10% fetal bovine serum (FBS; 10270106, Gibco, Thermo Fisher Scientific, Grand Island, NY, USA), and the cells were maintained at 37 °C in a humidified atmosphere containing 5% CO_2_. The FBoV-1 strain ZZ202401 (GenBank accession number: PX583168) was preserved in our laboratory. A mouse polyclonal antibody (pAb) anti-FBoV-1 VP2 protein was prepared and stored in our laboratory. CoraLite488-conjugated goat anti-mouse IgG (H+L) (SA00013-1) and goat anti-mouse IgG (SA00001-1), IgG1 (SA00012-1), IgG2a (SA00012-2), IgG2b (SA00012-3), and IgG3 (SA00012-5) secondary antibodies were purchased from Proteintech (Wuhan, China).

### 2.2. Construction of Recombinant Baculovirus

The VP2 gene was inserted into the *Bam*HI and *Hind*III restriction sites of the pFastBac1 vector to generate the recombinant plasmid pFastBac1-FBoV VP2, which was synthesized by GenScript Biotech (Nanjing, China). This recombinant vector was then transformed into the *E. coli* DH5α strain for amplification. The recombinant vector was verified by Sanger sequencing and then transformed into *E. coli* DH5α cells for amplification. The subsequent experimental procedures followed the guidelines provided in the Bac-to-Bac baculovirus expression system manual (Invitrogen, Carlsbad, CA, USA). Briefly, plasmids isolated from the transformed *E. coli* DH5α strain were re-transformed into the *E. coli* DH10Bac strain. Shuttle plasmids (rBacmid-VP2) were identified through Tn7 transposition and blue/white spot screening and extracted. Following this, Sf9 insect cells were transfected with the shuttle plasmids. After 3–4 days, the supernatants from the Sf9 insect cells were harvested, yielding the first generation of recombinant baculovirus (AcMNPV-VP2). The target genes in recombinant plasmids and recombinant baculovirus were confirmed by PCR using universal M13 primers designed according to the Bac-to-Bac baculovirus expression system (Invitrogen, Carlsbad, CA, USA) (forward primer: 5′-CCCACCATCGGGCGCGGATCCATGGAAAATGAAGTTGAGACGGCTGG-3′; reverse primer: 5′-CTAGTACTTCTCGACAAGCTTTCACAGTACTTTGTTGATTCCCCATTTT-3′).

### 2.3. Indirect Immunofluorescence Assay (IFA)

Recombinant baculoviruses were inoculated into Sf9 cells cultured in 24-well plates, with uninfected cells serving as controls. The cells were incubated at 27 °C for 48 h. Subsequently, they were fixed with 4% paraformaldehyde for 30 min. The fixative was then discarded, and 0.1% Triton X-100 was added for permeabilization for another 10 min. After washing three times with phosphate-buffered saline (PBS), the cells were blocked with PBS containing 10% FBS at 37 °C for 1 h. After additional washes with PBS, the cells were incubated with a polyclonal antibody anti-FBoV-1 VP2 protein (serving as the primary antibody), followed by incubation with CoraLite488-conjugated goat anti-mouse IgG as the secondary antibody. After washing with PBS, the cells were observed under an inverted fluorescence microscope (Olympus, Tokyo, Japan).

### 2.4. Expression and Purification of the VP2 Protein

We propagated the primary-generation baculovirus in Sf9 cells for 2 passages, then inoculated the third-passage baculovirus into Hi5 insect cells at a ratio of 1:100 (*v*/*v*) for recombinant protein expression. After 3 days, Hi5 cells infected with AcMNPV-VP2 were collected by centrifugation at 4000 rpm for 5 min to obtain the VP2 protein.

To purify the VP2 protein, Hi5 cells infected with AcMNPV-VP2 were resuspended in lysis buffer containing 20 mM HEPES and 50 mM NaCl (pH 8.0) and disrupted by sonication. The cell lysate was centrifuged, and the supernatant was collected for purification. The VP2 protein was initially purified using an anion-exchange chromatography column (17051010, Cytiva, Marlborough, MA, USA), followed by further purification by size-exclusion chromatography (SEC) using a HiLoad™ 16/600 Superose™ 6 pg column (29323952, Cytiva, Marlborough, MA, USA). The collected samples were subjected to SDS-PAGE, after which the protein gels were stained with Coomassie Brilliant Blue. After decolorization, images were collected using a gel imaging system (Bio-Rad, Hercules, CA, USA), and protein purity was analyzed using Image J 1.46r (Bethesda, Maryland, USA) software.

### 2.5. Western Blotting

Purified VP2 protein samples were separated by SDS-PAGE and transferred onto polyvinylidene difluoride (PVDF) membranes (ISEQ00010, Millipore, Darmstadt, Germany). The membranes were blocked with 5% non-fat milk for 1 h at room temperature and subsequently incubated overnight at 4 °C with a primary mouse anti-VP2 polyclonal antibody. After washing, the membranes were incubated with the corresponding HRP-conjugated secondary antibody for 1 h at room temperature. Protein bands were visualized using the Luminate Crescendo Western HRP Substrate (WBLUR0500, Millipore, Germany) and captured with a GE AI600 imaging system (GE, Boston, MA, USA).

### 2.6. Transmission Electron Microscope (TEM)

To verify whether the VP2 protein could self-assemble into VLPs, the purified VP2 protein was characterized by TEM. The sample was dropped on a carbon–formvar copper grid, and negative staining with phosphotungstic acid was performed. Excess stain was removed using filter paper, and the grids were dried at room temperature. The purified recombinant proteins were observed and photographed using a TEM (JEM-1400, Tokyo, Japan) at an accelerating voltage of 100 kV.

### 2.7. Dynamic Light Scattering (DLS)

To determine the particle size of the expressed protein, the sample was analyzed by DLS using a Zetasizer Nano ZS90 (Malvern Instruments, Malvern, UK). The purified protein was measured in triplicate, with 10 runs per measurement. The size distribution of VP2 nanoparticles in PBS was recorded.

### 2.8. Animal Immunization

All experimental procedures involving animals were reviewed and approved by the Institutional Animal Care and Use Committee of Henan Agricultural University (Zhengzhou, China) (approval number: HNND2024030711). The 6-week-old female BALB/c mice were purchased from the Liaoning Changsheng Biotechnology (Liaoning, China) and maintained under SPF conditions in accordance with the Guide for the Care and Use of Laboratory Animals and Henan Agricultural University ethical regulations. Mice were euthanized under isoflurane anesthesia followed by cervical dislocation. After a 1-week acclimation period, 30 female BALB/c mice were randomly divided into 5 groups, with 6 mice in each group. The groups were as follows: VP2, VP2+Alum, VP2+ISA 206, VP2+GEL 02, and PBS as the negative control. The immunization dose was 20 μg of VP2 protein per mouse. The VP2 protein was formulated with an aluminum adjuvant, Montanide™ ISA 206 VG (SEPPIC, Castres, France), or Montanide™ GEL 02 (SEPPIC, La Garenne-Colombes, France) at a 1:1 (*v*/*v*) ratio according to the manufacturers’ recommendations. Each mouse received 200 μL per immunization, and the mice in each group were immunized subcutaneously three times at 14-day intervals. Blood samples were collected weekly after immunization, and spleens were harvested at 42 days post-primary immunization for lymphocyte isolation. Three mice were randomly selected for flow cytometry and other tissue-based analyses, and the data presented represented these three biological replicates.

### 2.9. Enzyme-Linked Immunosorbent Assay (ELISA)

Serum was assayed for antibody titers and subtypes by ELISA. Briefly, the VP2 protein was diluted to 1 µg/mL using PBS (pH 7.2), added to ELISA plates at 100 µL/well, and incubated overnight at 4 °C. After 5 washes with PBST, the plates were sealed with 5% non-fat milk in PBS at 37 °C for 1 h. After washing with PBST, the serum samples were diluted with PBS, added to the wells, and incubated at 37 °C for 1 h. Thereafter, 100 μL of the goat anti-mouse secondary antibody IgG (1:2000), IgG1 (1:2000), IgG2a (1:2000), IgG2b (1:4000) or IgG3 (1:2000) was added and incubated at 37 °C for 1 h. The plates were washed five times with PBST after each incubation step. Subsequently, the 3,3′,5,5′-tetramethylbenzidine (TMB) substrate solution (100 μL/well; PR1200, Solarbio, Beijing, China) was added, and the plates were incubated in the dark at 37 °C for 10 min. The reaction was stopped by adding 2 M H_2_SO_4_ (50 μL/well; C1058, Solarbio, Beijing, China), and absorbance was measured at 450 nm (OD_450 nm_) using an enzyme meter (Thermo, Waltham, MA, USA). P/N (OD_450 nm_ of the sample to be measured/OD_450 nm_ of the negative control) ≥ 2.1 was used as the criterion for positive determination.

ELISA was also used to detect cytokines. Mouse IFN-γ, IL-2, IL-4 and IL-10 ELISA kits (JLW10967, JLW20256, JLW20266, JLW20242, Jonlnbio, Shanghai, China) were used for quantifying cytokines in serum. Serum dilution at a 1:10 ratio was used for the detection of IFN-γ, IL-2, IL-4 and IL-10. Absorbance was measured at OD_450 nm_. The concentrations of the cytokines were determined according to the standard curves.

### 2.10. Virus Neutralization Test (VNT)

The FBoV serum neutralization test was performed in 96-well cell culture plates. Briefly, serum samples were inactivated at 56 °C for 30 min before detection, serially two-fold diluted, and then mixed with an equal volume of the FBoV-1 strain ZZ202401 (200 TCID_50_/0.1 mL). After incubation at 37 °C for 1 h, the mixtures were added in quadruplicate to CRFK cell monolayers in 96-well cell culture plates. A serum-free virus mixture was included as a control. After incubation at 37 °C with 5% CO_2_ for 5 days, cells were fixed with 4% paraformaldehyde, and virus infection was detected by IFA. VNT titers were calculated as the reciprocal of the highest serum dilution that blocks 50% of viral infection, and the Reed–Muench method was used to further confirm the levels of the neutralizing antibody.

### 2.11. Splenic Index and Lymphocyte Proliferation Assay

At 42 days post-primary immunization, three mice from each group were randomly selected and euthanized under isoflurane anesthesia followed by cervical dislocation. The spleens of the mice in each group were harvested and weighed, and ratios were made according to the body weights of the mice. The splenic index was calculated as mouse spleen weight (mg)/mouse body weight (g). The mean value derived from 3 individuals was used as the final splenic index for each mouse. Splenic lymphocytes were subsequently isolated for lymphocyte proliferation assays.

For the splenic lymphocyte proliferation assay, the splenic lymphocytes of mice were isolated and seeded into 96-well plates at a density of 1 × 10^7^ cells/mL in an RPMI 1640 medium supplemented with 10% FBS. The cells were cultured at 37 °C with 5% CO_2_ and then stimulated with VP2 proteins at a final concentration of 10 µg/mL. Unstimulated cells were used as negative controls. After 48 h of incubation, the CCK-8 reagent (ZP328-3, ZOMANBIO, Beijing, China) was added to each well, followed by incubation for another 2 h at 37 °C. The OD value at 450 nm was read with a microplate reader (Thermo, Waltham, MA, USA). The stimulation index (SI) was calculated as follows: SI = (OD values of immunized groups − OD values of blank controls)/(OD values of negative controls − OD values of blank controls).

### 2.12. Preparation of Fluorescently Labeled Protein

To prepare fluorescently labeled proteins, Sulfo-Cyanine 5.5 NHS ester (D10020, DuoFluor, Wuhan, China) dissolved in dimethyl sulfoxide (DMSO) was added to VP2 proteins in PBS (pH 7.2) at a dye-to-protein molar ratio of 10:1. The mixture was gently mixed and allowed to react at 4 °C for 4 h. Unconjugated free dye was removed by dialysis using a dialysis bag with a molecular weight cutoff of 10 kDa. During dialysis, the dialysis buffer was replaced every 4 h for a total of three times. The Sulfo-Cyanine 5.5 NHS ester-labeled VP2 protein, designated as Cy5.5-VP2 to distinguish it from unlabeled VP2, was identified using inverted fluorescence microscopy.

### 2.13. Activation and Maturation of Bone Marrow-Derived Dendritic Cells (BMDCs)

BMDC Extraction: The femur and tibia of a euthanized BALB/c mouse were extracted under sterile conditions, and the bone marrow cells were obtained by rinsing with a Roswell Park Memorial Institute (RPMI) 1640 medium (31800, Solarbio, Beijing, China). The collective bone marrow cells were filtered using a 70-mesh sieve and then centrifuged at 500 g for 10 min. The cells were resuspended in 2 mL of red blood cell lytic buffer (R1010, Solarbio, Beijing, China) for 3 min. The suspension was centrifuged, the sediment was washed with RPMI 1640, and the cells were resuspended in an RPMI 1640 complete medium consisting of 10% FBS, 1% penicillin–streptomycin (P1400, Solarbio, Los Angeles, LA, USA), 100 ng/mL GM-CSF (Eg0512, Proteintech, Wuhan, China), and 50 ng/mL IL-4 (Eg3750, Proteintech, Wuhan, China). The cells were cultured at 37 °C with 5% CO_2_, and half of the medium was replaced with fresh medium on days 3 and 5. On day 7, suspended cells were collected for subsequent experiments.

BMDCs were inoculated in 24-well plates at a density of 5 × 10^5^ cells per well and cultured overnight. VP2, VP2+Alum, VP2+ISA 206, or VP2+GEL 02 was added for 24 h of incubation, and PBS-treated cells were used as a negative control. After 24 h, BMDCs were harvested and washed, and cells were labeled with Brilliant Violet 421™ anti-CD11c, APC anti-CD80, PE anti-CD86, and PE anti-I-A/I-E for 30 min at 4 °C under darkness. These antibodies were obtained from BioLegend (117343, 104713, 105007, and 107607; BioLegend, San Diego, CA, USA). Cells were washed with PBS and analyzed by CytoFLEX flow cytometry (Beckman Coulter, Brea, CA, USA).

### 2.14. Cell Cytotoxicity Test

DC2.4 cells or BMDCs were inoculated into 96-well culture plates at a density of 1 × 10^4^ cells/well and cultivated overnight at 37 °C in a humidified incubator with 5% CO_2_. The culture medium was then replaced with an RPMI 1640 medium supplemented with 10% FBS and containing VP2, VP2+Alum, VP2+ISA 206, or VP2+GEL 02. After incubation for 24 h, cell viability was measured using cell counting kit 8 (CCK-8; ZP328-3, ZOMANBIO, Beijing, China) according to the manufacturer’s instructions.

### 2.15. Detection of VLP Uptake by DCs

DC2.4 cells or BMDCs were seeded into 24-well plates at a density of 5 × 10^5^ cells/well and cultured overnight. The cells were then treated with Cy5.5-VP2, Cy5.5-VP2+Alum, Cy5.5-VP2+ISA 206, or Cy5.5-VP2+GEL 02. After 12 h of incubation, the cells were harvested and washed three times with PBS, assayed using a CytoFLEX flow cytometer, and analyzed using FlowJo software (version 10.9.0).

### 2.16. In Vivo Imaging in Mice

Fifteen 8-week-old female BALB/c mice were randomly divided into five groups of three mice each. Mice in each group were subcutaneously injected in the right leg with Cy5.5-VP2 (control), where the amount of the Cy5.5-VP2 antigen was consistent. The fluorescence intensity of each sample was tested in vitro to ensure that the initial fluorescence intensity of each group was similar. For *in vivo* imaging, mice were anesthetized by intraperitoneal injection of sodium pentobarbital (50 mg/kg), and the biodistribution of fluorescent antigens was monitored at different time points using an IVIS Lumina III small-animal *in vivo* imaging system (PerkinElmer, Shelton, CT, USA). At 96 h post-injection, three mice from each group were euthanized under isoflurane anesthesia followed by cervical dislocation. The mice were subsequently necropsied, and spleens were collected for *ex vivo* fluorescence imaging to evaluate antigen biodistribution. Fluorescence radiation efficiency was quantified using the self-contained real-time imaging data analysis software.

### 2.17. Immunohistochemistry (IHC)

The spleens of mice were isolated 14 days after three immunizations and fixed with paraformaldehyde, and paraffin sections were made. The spleen-tissue slides were deparaffinized, treated with 3% H_2_O_2_ for 10 min, autoclaved in 10 mM citric sodium (pH 6.0) for 30 min to unmask antigens, rinsed in PBS and then incubated with anti-Ki67 rabbit mAb (GB151499, Servicebio, Wuhan, China) at 4 °C overnight, followed by incubation with an HRP-labeled anti-rabbit IgG secondary antibody (G1302, Servicebio, Wuhan, China) for 1 h at room temperature. Finally, 3,3-diaminobenzidine tetrahydrochloride was used as a coloring reagent, and hematoxylin was used as a counterstain for nuclei. Sections were scanned using a digital pathology slice scanner (LG-S80, Servicebio, Wuhan, China) and images were observed and recorded by Saiviewer browsing and analysis software (Saiviewer, Servicebio, Wuhan, China).

### 2.18. In Vivo Safety Study

Next, 6-week-old female BALB/c mice were injected subcutaneously in the back with the VP2, VP2+Alum, VP2+ISA 206, or VP2+GEL 02 vaccines (VP2 protein 20 μg), and the PBS-injected group was used as a control. Three mice in each group were injected every 14 days. On day 42, the blood and organs (heart, liver, spleen, lungs and kidneys) of the mice were collected. Blood biochemical parameters (ALT, AST, ALP, BUN, CREA, and LDH) were determined using a biochemical reagent kit (BC1555, BC1565, BC2145, BC1535, BC4915, and BC0685; Solarbio, Beijing, China). Organs were fixed, paraffin embedded, sectioned and stained with hematoxylin and eosin (H&E).

### 2.19. Flow Cytometry (FCM) Analysis

At 42 days post-primary immunization, three mice from each group were randomly selected and euthanized under isoflurane anesthesia followed by cervical dislocation. The spleens were aseptically harvested and homogenized through a 70-mesh sieve to prepare single-cell suspensions, and the cells were adjusted to a concentration of 1 × 10^7^/mL. CD4^+^ T cells, CD8^+^ T cells, germinal center (GC) B cells, Tfh cells, plasma cells, and memory B cells were analyzed by the flow cytometer (Beckman, Brea, CA, USA). For detection of the percentages of proliferating B cells and T cells, the splenocytes were stimulated with specific antigens. Cell surface antigens were stained for 30 min at 4 °C. The antibodies used for flow cytometry were as follows: FITC anti-CD3, PE anti-CD4, APC anti-CD8, Brilliant Violet 421™ anti-CD19, FITC anti-IgD, PE anti-CD69, APC anti-CD3, APC anti-B220, Brilliant Violet 421™ anti-CD3, APC anti-CD4, APC anti-CD95 (Fas), PE anti-GL7, Brilliant Violet 421™ anti-CD185 (CXCR5), PE anti- CD279 (PD-1), PE anti-CD138 and APC anti-CD38. They were all bought from Biolegend (100204, 100408, 100712, 115549, 405703, 104507, 100236, 103211, 100227, 100411, 152603, 144607, 145511, 109103, 142503, and 102711; BioLegend, San Diego, CA, USA). The data were analyzed by Flowjo V10.9.0 (Ashland, OR, USA).

### 2.20. Statistical Analysis

Statistical analyses were performed using GraphPad Prism version 8.0 (San Diego, CA, USA). Data are presented as the mean ± standard deviation (SD). Comparisons among multiple groups at a single time point were performed using one-way analysis of variance (one-way ANOVA) followed by Tukey’s multiple comparison test. For experiments involving multiple groups and multiple time points, two-way ANOVA followed by Tukey’s multiple comparison test was used. A *p*-value < 0.05 was considered statistically significant (*: *p* < 0.05, **: *p* < 0.01, ***: *p* < 0.001, and ns: not significant).

## 3. Results

### 3.1. Preparation and Characterization of FBoV VP2 VLPs

**Figure 1:**
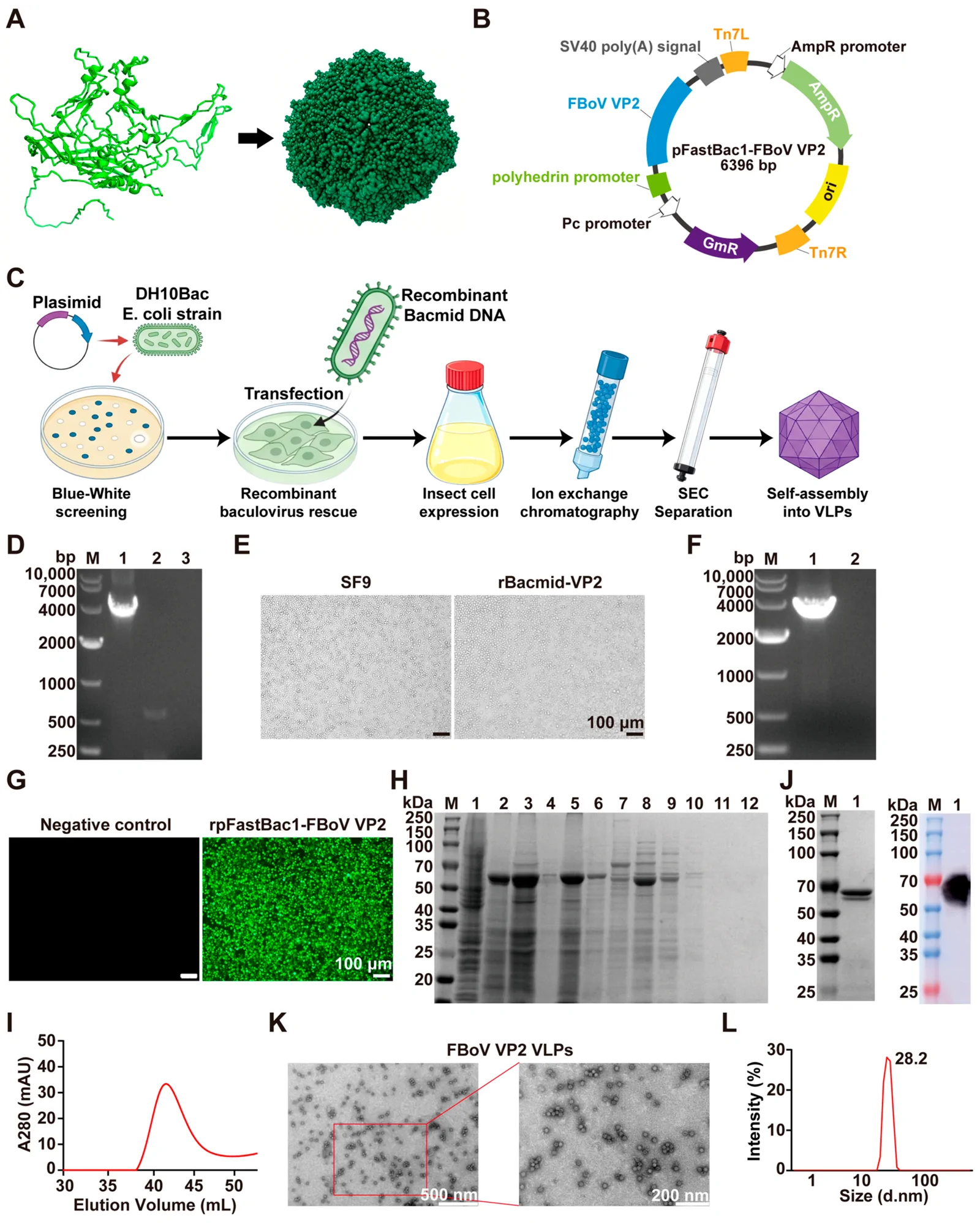
Preparation and structural characterization of FBoV VP2 VLPs. (**A**) The monomeric structure of the FBoV VP2 protein was predicted using AlphaFold3, and its self-assembly into VLPs was further modeled. (**B**) Construction map of the pFastbac1-FBoV VP2 plasmid. (**C**) Expression and purification process of the FBoV VP2 protein. The VP2 protein was expressed using a baculovirus expression system. The recombinant bacmid rBacmid-VP2 was obtained through blue-white screening and then transfected into Sf9 cells to rescue the recombinant baculovirus AcMNPV-VP2. Hi5 cells were subsequently infected with AcMNPV-VP2 for VP2 protein expression and extraction. The clarified cell lysate was purified by anion-exchange chromatography followed by size-exclusion chromatography. (**D**) Identification of the recombinant bacmid rBacmid-VP2 by PCR. Lane M: marker; Lane 1: recombinant plasmid Bacmid-VP2; Lane 2: wild-type bacmid plasmid; Lane 3: negative control. (**E**) Normal sf9 cells (**left**) and sf9 cells transfected with the recombinant plasmid Bacmid-VP2 (**right**). (**F**) PCR analysis confirmed the successful rescue of the recombinant baculovirus AcMNPV-VP2. Lane M: marker; Lane 1: recombinant baculovirus AcMNPV-VP2; Lane 2: negative control. (**G**) Identification of the VP2 protein by IFA. Anti-FBoV VP2 polyclonal antibodies were used for the detection of IFA. (**H**) Identification of primary purified VP2 proteins by anion exchange chromatography. Lane M: marker; Lane 1: normal Hi5 cells; Lane 2: Hi5 cells infected with AcMNPV-VP2; Lane 3: ultrasonic disruption of the cell supernatant from Hi5 cells infected with AcMNPV-VP2; Lane 4: ultrasonic disruption of cell precipitation in Hi5 cells infected with AcMNPV-VP2; Lane 5: flow through after binding of the supernatant to the anionic filler Q Sepharose Fast Flow; Lanes 6-12: Fractions of the primary purified VP2 protein eluted with an elution buffer containing 0, 0.1, 0.2, 0.3, 0.5, 0.8, and 1 M NaCl, respectively. (**I**) SEC elution profile of the purified recombinant VP2 proteins on a HiLoad™ 16/600 Superose™ 6 pg column. (**J**) Identification of the purified VP2 protein. Lane M: marker; Lane 1: purified VP2 protein. (**K**) TEM images of purified VP2 VLPs. Scale bar, 500 nm and 200 nm. (**L**) DLS analysis of purified VP2 VLPs.

FBoV particles exhibit a typical icosahedral structure with a diameter of approximately 23–28 nm. The VP2 protein of FBoV is a key structural component, and based on the structure of bocaviruses, VP2 can self-assemble into 60-mer spherical VLPs (Figure 1A). We utilized the baculovirus expression system to express FBoV VP2 proteins, as shown in Figure 1C. The pFastBac1-FBoV VP2 plasmid (Figure 1B) was successfully constructed. The recombinant baculovirus AcMNPV-VP2 was successfully generated, as verified by PCR (Figure 1D). To generate the recombinant baculovirus, the recombinant plasmid rBacmid-VP2 was transfected into SF9 cells, and after 96 h of transfection, Sf9 cells exhibited typical cytopathic effects (CPEs), including cell enlargement, rounding, and detachment, indicating successful rescue of the recombinant baculovirus AcMNPV-VP2 (Figure 1E). The AcMNPV-VP2 baculovirus was continuously passaged three times to obtain high-titer viral strains. The presence of the VP2 gene in AcMNPV-VP2 was confirmed by PCR (Figure 1F). IFA was performed to detect the VP2 protein, and the results confirmed the specificity of the VP2 protein (Figure 1G). The VP2 protein was successfully expressed in Hi5 cells and purified by anion exchange chromatography and size-exclusion chromatography. SDS-PAGE and Western blot analysis confirmed the successful purification of the recombinant VP2 protein with high purity (Figure 1H–J). Based on analysis with Image J software, the purity of the FBoV-VP2 protein was 86.7%. TEM and DLS analyses demonstrated that purified VP2 proteins formed uniform spherical nanoparticles with an average diameter of approximately 28 nm, confirming the successful assembly of VP2 VLPs (Figure 1K,L). Taken together, these findings demonstrate that VP2 proteins can efficiently assemble into stable VLPs and that the insect cell expression system enables high-yield production of VLPs.

### 3.2. FBoV VP2 VLP Vaccines Induce Potent and Durable Humoral Immune Responses in Mice

**Figure 2:**
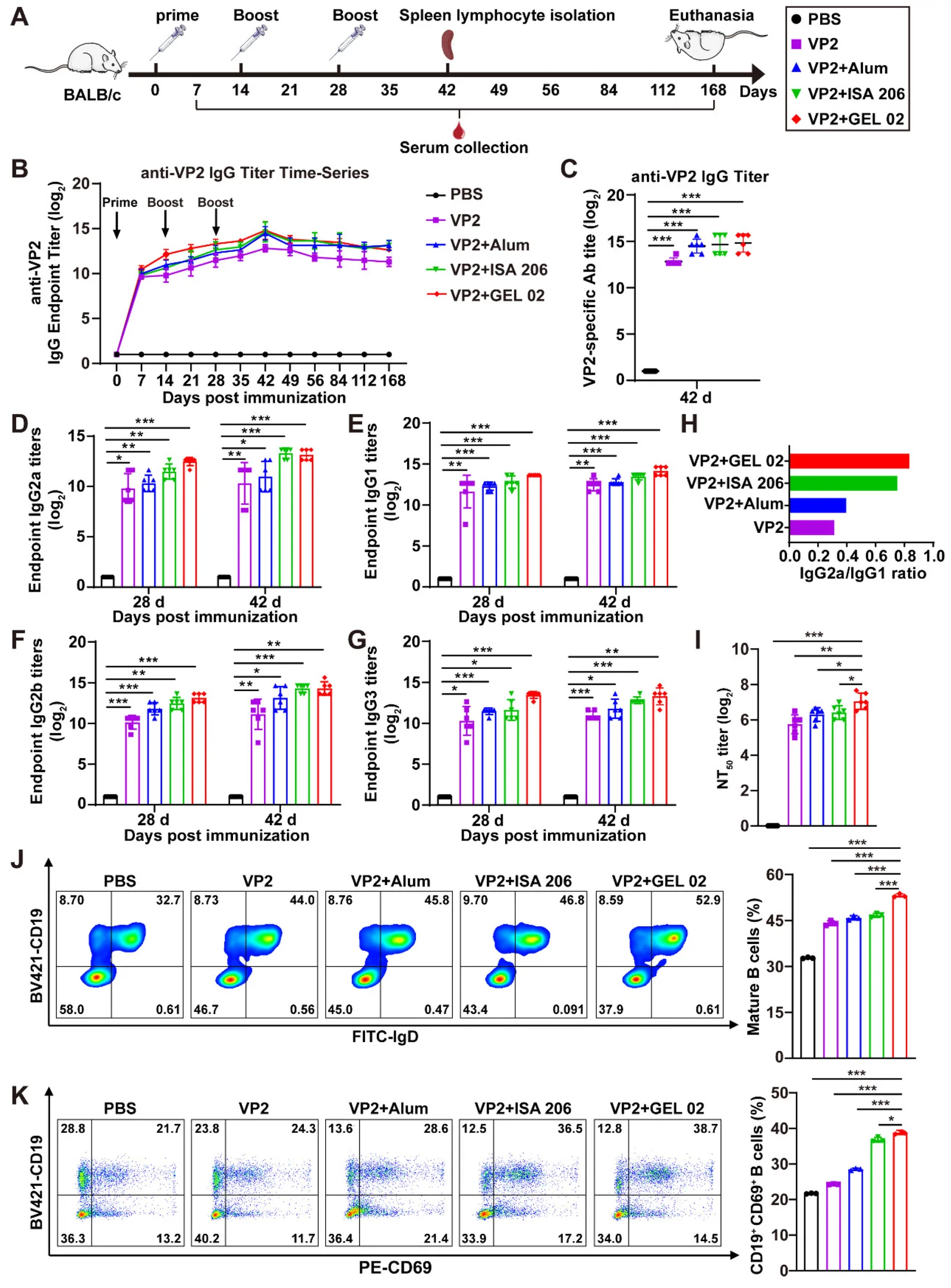
Humoral immune responses induced in immunized mice. (**A**) Schedule of mouse immunization and sample collection. BALB/c mice were immunized thrice at 14-day intervals, and the legend on the right lists all immunized groups (*n* = 6). (**B**) VP2-specific IgG titers in immunized BALB/c mice were measured by ELISA at the indicated time points and plotted as a time-course curve. (**C**) IgG antibody titers in serum samples collected on day 42 were determined by serial dilution and expressed as the reciprocal of the endpoint serum dilution. (**D**–**G**) Antigen-specific IgG2a (**D**), IgG1 (**E**), IgG2b (**F**), and IgG3 (**G**) titers were determined in each immunized group. Serum samples collected from mice on days 21 and 42 were analyzed. (**H**) IgG subclass profile, shown as the IgG2a/IgG1 ratio, on day 42 after the initial immunization. (**I**) Anti-FBoV neutralizing antibody titers. (**J**,**K**) Percentage of mature B cells (CD19^+^ IgD^+^) (**J**) and activated B cells (CD19^+^ CD69^+^) (**K**) in splenocytes detected by flow cytometry (*n* = 3). *: *p* < 0.05, **: *p* < 0.01, and ***: *p* < 0.001.

To evaluate the immunogenicity of the FBoV VP2 VLP vaccines, VP2-specific antibody responses were monitored after immunization (Figure 2A). The highest antibody levels were observed in all immunized groups on day 42 after the first immunization, and a high level of the VP2-specific IgG antibodies was still detectable on day 168 (Figure 2B). Compared with the other groups, the VP2+GEL 02 group induced the highest levels of VP2-specific IgG antibodies on day 42 after the first immunization (Figure 2C). To further explore the patterns of the humoral response induced by VLP vaccines, we analyzed the FBoV VP2-specific IgG subtypes by indirect ELISA on days 28 and 42. The results revealed that the VP2-specific IgG2a, IgG1, IgG2b and IgG3 titers are significantly higher in all immunized groups and that the IgG2a, IgG1, IgG2b and IgG3 titers of the VP2-GEL 02 group were higher than those of the other groups (Figure 2D–G). IgG1 and IgG2a are used as markers of the Th1 response and the Th2 response, respectively, and their ratio reflects the state of the antibody response. Meanwhile, the IgG2a/IgG1 ratio remained less than one throughout the immune process, indicating a predominant Th2-mediated antibody response (Figure 2H).

In order to investigate the anti-FBoV-1-neutralizing antibody titers in mice, the levels of the neutralizing antibody were determined using the serum sample collected 42 days post-primary immunization. All immunized groups elicited high levels of neutralizing antibodies, with the VP2+GEL 02 group inducing significantly higher levels than the other groups (Figure 2I). Subsequently, the frequencies of mature and activated B cells in mice were analyzed by flow cytometry. The results showed that the proportions of mature B cells and CD19^+^ CD69^+^ B cells were significantly increased in all immunized groups, with the VP2+GEL 02 group showing significantly higher levels than the other groups (Figure 2J,K).

### 3.3. Detection of Splenic Lymphocyte Proliferation

**Figure 3:**
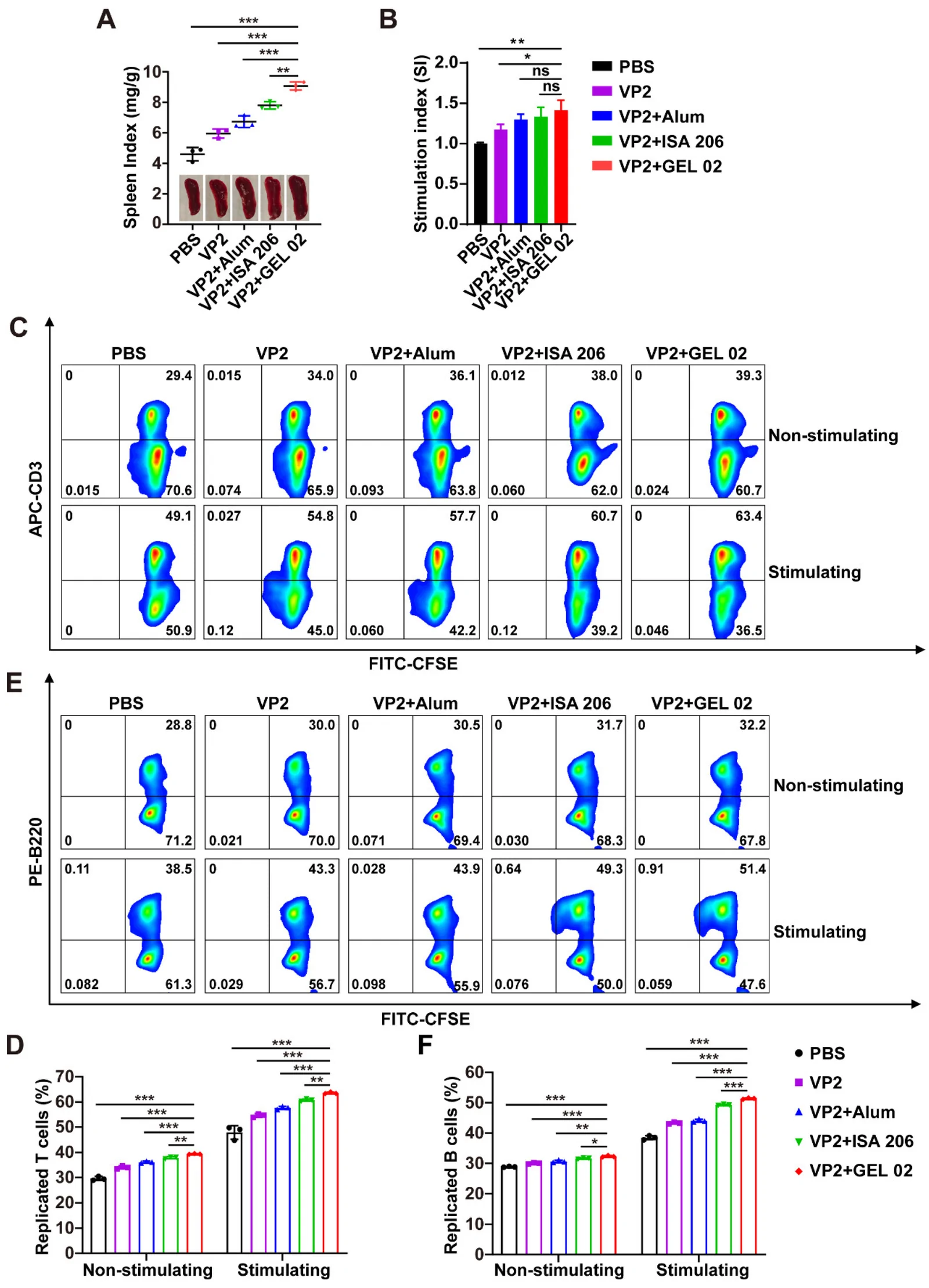
Splenic lymphocyte proliferation. (**A**) Splenic index. Spleens were harvested from mice in each group on day 42 after the first immunization and weighed. The splenic index was calculated as spleen weight (mg)/body weight (g) (*n* = 3). (**B**) Splenic lymphocyte proliferation assay (*n* = 3). (**C**,**D**) Percentage of proliferation of T lymphocytes in splenocytes detected by flow cytometry (*n* = 3). (**E**,**F**) Percentage of proliferation of B lymphocytes in splenocytes detected by flow cytometry (*n* = 3). *: *p* < 0.05, **: *p* < 0.01, ***: *p* < 0.001, and ns: not significant.

The splenic index reflects to some extent the strength of the immune response. The spleens of mice in each immunization group were observed and weighed, and the splenic index of the VP2+GEL 02 immunization group was significantly higher than that of the other groups (Figure 3A). At day 42 of the first immunization, the splenocytes of mice were isolated for lymphocyte proliferation detection using CCK-8 (Figure 3B). After being stimulated with the VP2 protein, the stimulation index of lymphocyte proliferation in the VP2+GEL 02 group was significantly higher than that of the PBS and VP2 groups, and there were no significant differences among the three adjuvant groups. Meanwhile, splenic lymphocytes were labeled with CFSE and stimulated with VP2 for 24 h. The proliferation of B and T lymphocytes was analyzed by flow cytometry (Figure 3C–F). The results showed that all immunized groups significantly promoted the proliferation of B and T lymphocytes, with the VP2+GEL 02 group exhibiting significantly higher proliferative responses than the other groups. These results indicate that the FBoV VP2 VLP vaccine can enhance immune responses in mice, particularly in the VP2+GEL 02 group.

### 3.4. Induction of Cellular Immune Responses by FBoV VP2 VLP Vaccines in Mice

**Figure 4:**
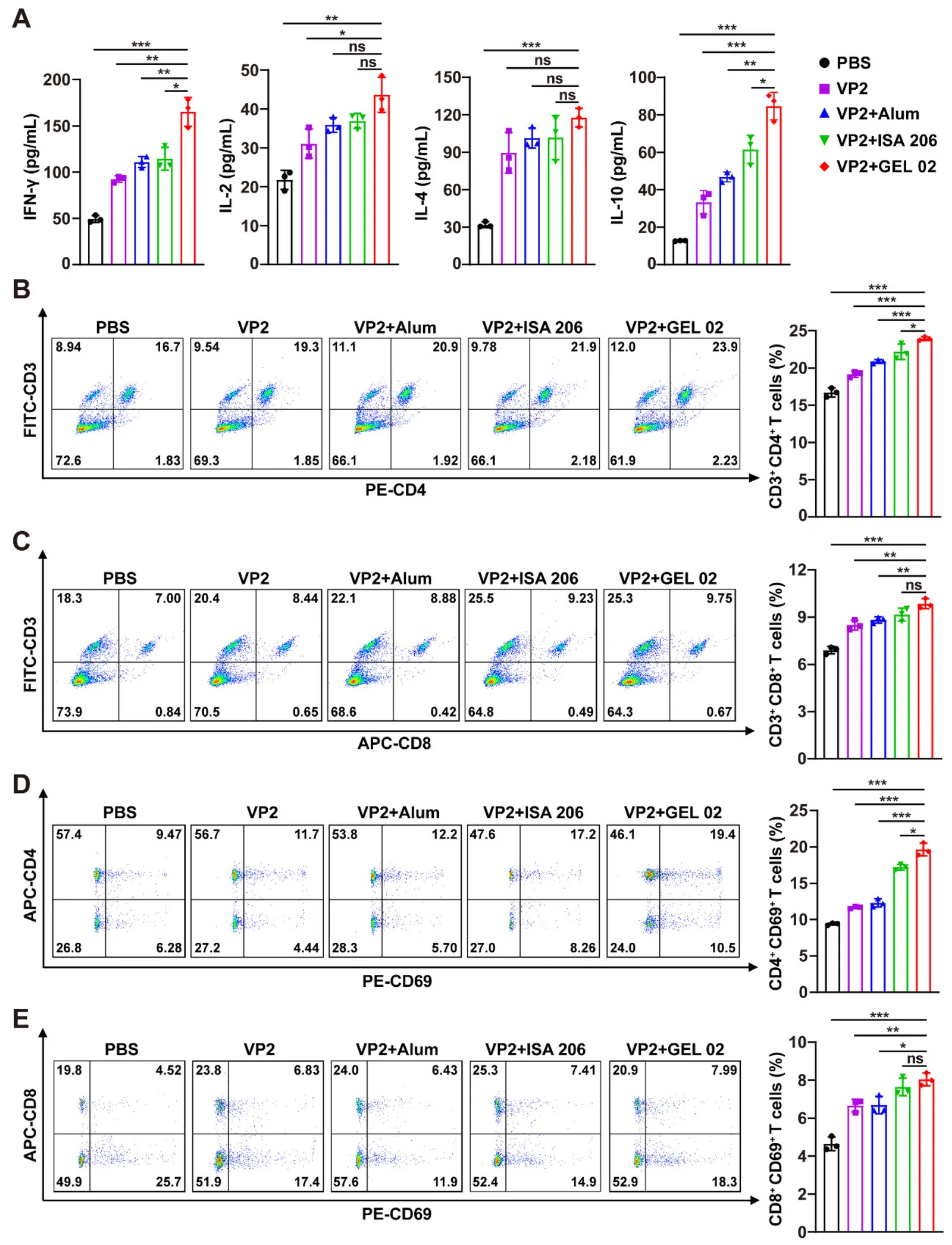
Cellular immune responses. (**A**) Serum samples were collected from the immunized mice at day 42. IFN-γ, IL-2, IL-4 and IL-10 were detected through ELISA. (**B**,**C**) The percentages of CD4^+^ T and CD8^+^ T cells in the splenocytes were analyzed through flow cytometry. (**D**,**E**) The percentages of CD4^+^ CD69^+^ T cells and CD8^+^ CD69^+^ T cells in the splenocytes were analyzed through flow cytometry. *: *p* < 0.05, **: *p* < 0.01, ***: *p* < 0.001, and ns: not significant.

Serum cytokine levels were measured using ELISA kits on day 42 after the first immunization. Compared with the PBS group, all immunized groups showed elevated levels of IFN-γ, IL-2, IL-4, and IL-10 (Figure 4A). Among the serum samples, the levels of IFN-γ and IL-10 induced by the VP2+GEL 02 group were significantly higher than those induced by the other groups, whereas no significant differences in IL-2 or IL-4 levels were observed among the immunized groups. Overall, the VP2+GEL 02 group induced the highest cytokine levels among all groups, although the difference was not significant.

Subsequently, cellular immune responses induced by the FBoV VP2 VLP vaccine in mice were further evaluated by quantifying CD4^+^ T and CD8^+^ T cells using flow cytometry. Compared with the PBS group, the percentages of CD4^+^ T and CD8^+^ T cells were increased in all immunized groups (Figure 4B,C). The percentage of CD4^+^ T cells was significantly higher in the VP2+GEL 02 group than in the other groups. For CD8^+^ T cells, the VP2+GEL 02 group showed significantly higher percentages than the other groups, except for the VP2+ISA 206 group. In addition, activated CD4^+^ T and CD8^+^ T cells were identified by flow cytometry (Figure 4D,E). The results showed that the percentage of CD4^+^ CD69^+^ T cells was significantly higher in the VP2+GEL 02 group than in the other groups. Similarly, the percentage of CD8^+^ CD69^+^ T cells was significantly higher in the VP2+GEL 02 group than in the other groups, except for the VP2+ISA 206 group. Overall, these results indicate that the FBoV VP2 VLP vaccine enhances cellular immune responses in mice, particularly when formulated with the aqueous GEL 02 adjuvant, although the difference was not significant.

### 3.5. In Vivo Safety of the FBoV VP2 VLP Vaccine

**Figure 5:**
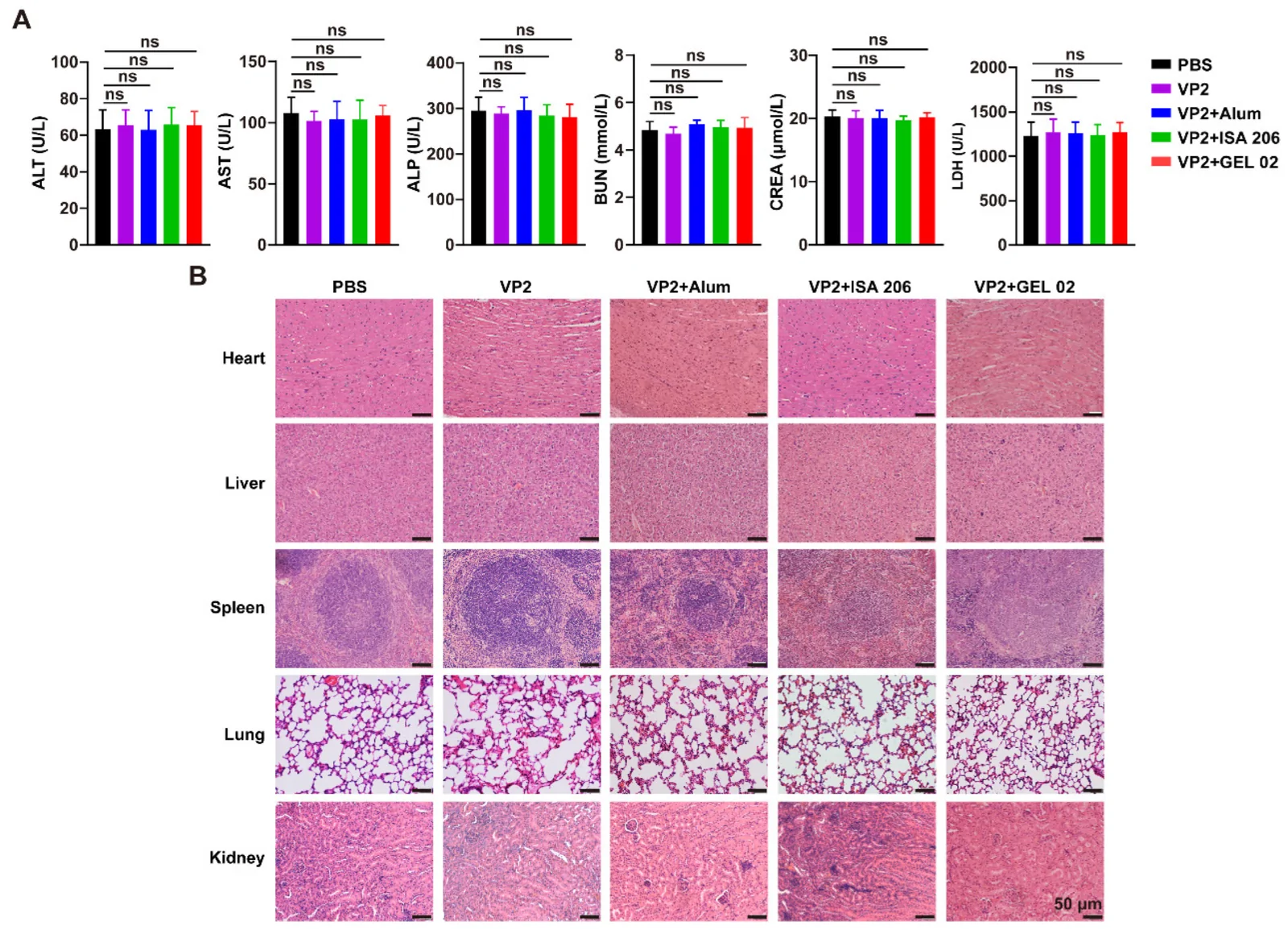
Biosafety of the FBoV VP2 VLP vaccine *in vivo*. (**A**) Levels of biochemical indicators in serum (ALT, AST, ALP, BUN, CREA and LDH) for mice after vaccination (*n* = 3). ns, not significant. (**B**) Representative histological pictures of major organs stained with H&E. Scale bar, 50 μm.

Low cytotoxicity and favorable biosafety are essential prerequisites for the potential clinical application of vaccines. To evaluate the biosafety of the FBoV VP2 VLP vaccine, blood samples and major organs, including the heart, liver, spleen, lungs, and kidneys, were collected from mice immunized with the FBoV VP2 VLP vaccine for serum biochemical analysis and histopathological evaluation. Compared with the PBS group, all immunized groups showed no significant differences in serum biochemical parameters, including alanine aminotransferase (ALT), aspartate aminotransferase (AST), alkaline phosphatase (ALP), blood urea nitrogen (BUN), creatinine (CREA), and lactate dehydrogenase (LDH), and all values remained within the normal ranges (Figure 5A). In addition, hematoxylin and eosin (H&E) staining of major organs revealed no obvious pathological changes (Figure 5B). These results indicate that the FBoV VP2 VLP vaccine exhibits a favorable biosafety profile.

### 3.6. In Vitro Cellular Uptake Analysis of FBoV VP2 VLPs and Promotion of BMDC Maturation

**Figure 6:**
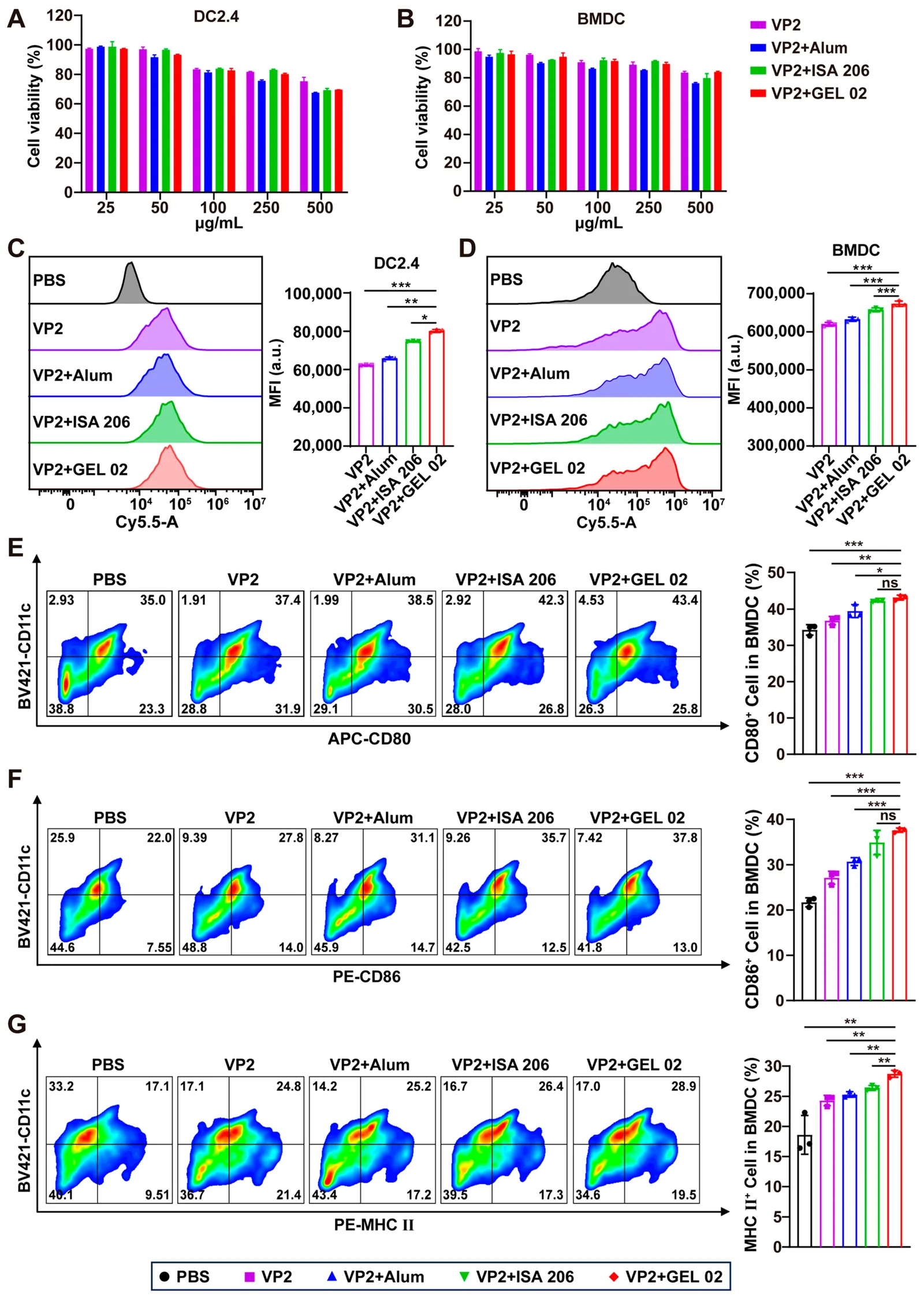
Cytotoxicity and cellular uptake promote the activation and maturation of BMDCs of FBoV VP2 VLPs in vitro. Cell viability of DC2.4 cells (**A**) and BMDCs (**B**) after incubation with different concentrations of FBoV VP2 VLPs for 24 h (*n* = 3). The cellular uptake of FBoV VP2 VLPs by DC2.4 cells (**C**) and BMDCs (**D**) was examined by flow cytometry (*n* = 3). (**E**–**G**) FCM analysis of BMDC maturation markers, including antigen delivery (MHC II) (**G**) and costimulatory molecule (CD80 and CD86) (**E**,**F**) expression (*n* = 3). *: *p* < 0.05, **: *p* < 0.01, ***: *p* < 0.001, and ns: not significant.

The cytotoxicity of different VP2 VLP formulations was evaluated in DC2.4 cells and BMDCs. All formulations showed favorable biocompatibility, with cell viability remaining above 70% even at high concentrations, indicating their low cytotoxicity and excellent biocompatibility. (Figure 6A,B). Effective antigen uptake by DCs was a critical step in the induction of potent immune responses. We investigated the cellular uptake of VP2 by DC2.4 cells and BMDCs. To evaluate nanovaccine uptake by APCs, VP2 was labeled with Cy5.5, and the percentage of Cy5.5-positive cells was subsequently analyzed by flow cytometry (Figure 6C,D). The results showed that the addition of adjuvants significantly enhanced antigen uptake by APCs, with the VP2+GEL 02 group exhibiting significantly higher uptake efficiency than the other groups. The ability of VP2 VLP formulations to activate dendritic cells was further evaluated by analyzing the expression of maturation markers, including MHC-II, CD80, and CD86. The results showed that, compared with the control group, the FBoV VP2 VLP vaccine significantly upregulated the expression of CD80, CD86, and MHC-II, indicating the induction of DC activation (Figure 6E–G). Among the immunized groups, the VP2+GEL 02 group showed significantly higher MHC-II expression than the other groups.

### 3.7. Study on the Biodistribution of the FBoV VP2 VLP Vaccine in Mice

**Figure 7:**
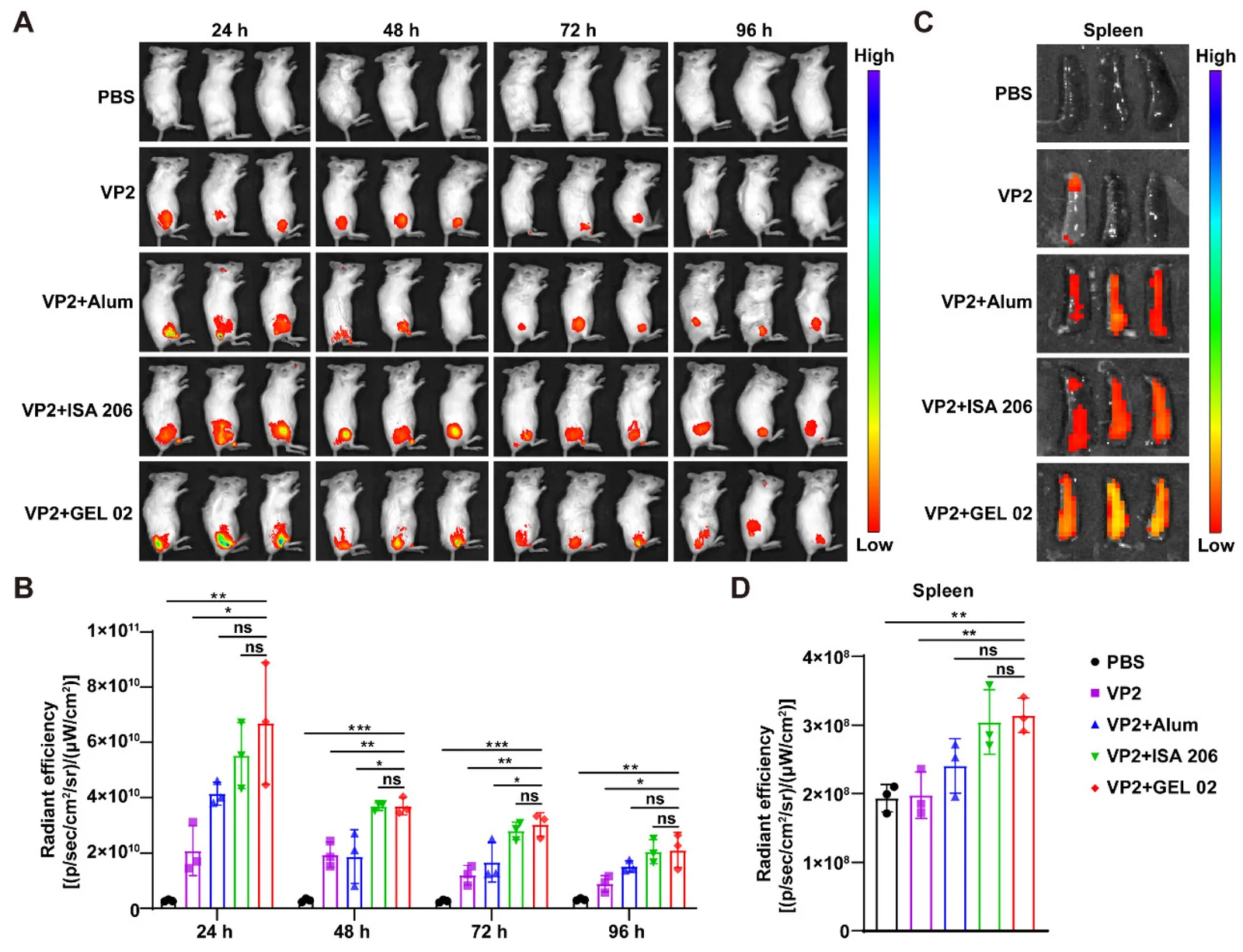
Biodistribution of the FBoV VP2 VLP vaccine in mice. (**A**) Biodistribution of Cy5.5-VP2 in mice from the different injection groups was analyzed at different time points using an IVIS spectrum system (*n* = 3). PBS-injected mice were used as negative controls. (**B**) Quantification of *in vivo* fluorescence radiation efficiency at each time point using real-time imaging data analysis software (*n* = 3). (**C**) Mice were necropsied 96 h after injection, and spleens were collected for *ex vivo* fluorescence imaging (*n* = 3). (**D**) Fluorescence efficiency in the spleen at 96 h after injection (*n* = 3). *: *p* < 0.05, **: *p* < 0.01, ***: *p* < 0.001, and ns: not significant.

To study antigen retention and distribution *in vivo*, we probed the transport kinetics of FBoV VP2 VLPs by *in vivo* imaging. First, local reactions at the injection site were evaluated by formulating Cy5.5-labeled VP2 with the Alum, ISA 206, or GEL 02 adjuvant and subcutaneously injecting the formulations into the right hind limb of BALB/c mice. As shown in Figure 7A, the fluorescent signal area and intensity at the injection site gradually decreased over time. In the free VP2 group, the fluorescence signal almost completely disappeared after 96 h. In contrast, the fluorescence signals in the Cy5.5-VP2+Alum, Cy5.5-VP2+ISA 206, and Cy5.5-VP2+GEL 02 groups decreased after 24 h but remained detectable at 96 h. At this time point, the fluorescence intensity in the Cy5.5-VP2+GEL 02 group remained higher than that in the other groups, although the difference was not significant (Figure 7B). At 96 h after injection, spleens were collected from mice in each group for *ex vivo* imaging (Figure 7C). Compared with the free VP2 group, mice injected with adjuvant-formulated VP2 showed stronger fluorescence signals in the spleen, particularly in the VP2+GEL 02 group, although the difference was not significant (Figure 7D). These results indicate that the addition of adjuvants can prolong antigen retention *in vivo*, establish an antigen depot at the injection site, and facilitate antigen delivery to secondary lymphoid organs. The formation of an antigen depot may enhance vaccine efficacy by prolonging antigen availability, regulating antigen release, extending antigen persistence, and recruiting nearby immune cells.

### 3.8. Promotion of GC Formation by the Fbov VP2 VLPS Vaccine

**Figure 8:**
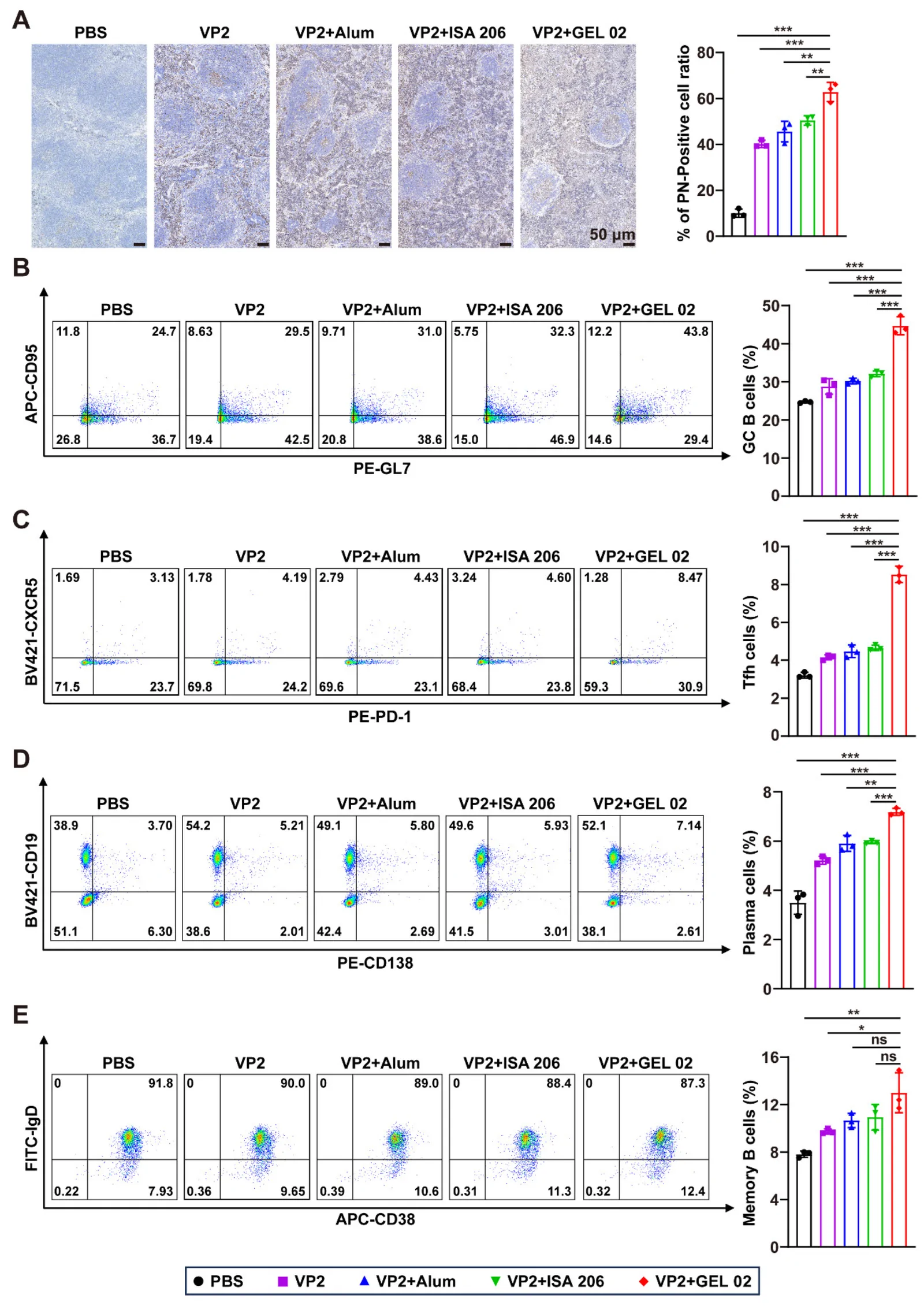
Promotion of GC formation by the FBoV VP2 VLP vaccine. (**A**) Representative images of IHC Ki67 staining of sections of spleens from each group. The proportion of positive cells was calculated using quantitative analysis software (*n* = 3). Scale bar: 50 μm. (**B**–**E**) The percentages of GC B cells (**B**), Tfh cells (**C**), plasma cells (**D**), and memory B cells (**E**) in different immunized groups were analyzed by flow cytometry. *: *p* < 0.05, **: *p* < 0.01, ***: *p* < 0.001, and ns: not significant.

GCs are a hallmark of T-cell-dependent humoral immune responses and are essential for the generation of potent antibody responses. GCs usually form in secondary lymphoid organs following infection or vaccination-induced antigen stimulation. Activated B cells rapidly proliferate within GCs. Therefore, Ki67 was used as a marker of proliferating and dividing cells to evaluate GC formation. IHC analysis of a draining spleen showed that immunization markedly promoted the formation of GC regions (Figure 8A). Quantitative analysis further showed that the proportion of Ki67-positive signals was significantly increased after immunization, particularly in the VP2+GEL 02 group, which exhibited significantly higher Ki67 positivity than the other groups (Figure 8A).

To further evaluate the generation and differentiation of immune cells, we measured the proportions of GC B cells, Tfh cells, plasma cells, and memory B cells among splenic lymphocytes after immunization. Flow cytometry analysis showed that the changes in Tfh cell frequencies were consistent with those observed for GC B cells (Figure 8B,C). After immunization, the proportions of GC B cells and Tfh cells were significantly higher in the VP2+GEL 02 group than in the other groups, indicating enhanced B-cell proliferation and differentiation. For plasma cells, all immunized groups showed increased proportions after immunization, with the VP2+GEL 02 group exhibiting significantly higher levels than the other groups (Figure 8D). Memory B cells also showed an increasing trend after immunization, particularly in the VP2+GEL 02 group, although no significant differences were observed between the VP2+Alum and VP2+ISA 206 groups (Figure 8E). These results indicate that the FBoV VP2 VLP vaccine effectively promotes the formation of spleen GCs and significantly enhances GC B-cell responses.

## 4. Discussion

FBoV is an emerging enteric pathogen in cats, and the lack of available vaccines highlights the need for effective preventive strategies. In this study, we successfully developed a baculovirus-expressed FBoV VP2 VLP vaccine and systematically evaluated its immunogenicity and immune activation mechanisms in mice. The results demonstrated that VP2 VLPs induced strong humoral and cellular immune responses, promoted dendritic cell activation, enhanced germinal center formation, and generated functional neutralizing antibodies. Previous studies have shown that FBoV-1 is the subtype most frequently detected in coinfections with other viral pathogens. FBoV can be classified into three subtypes, among which genetically diverse FBoV-1 and FBoV-2 strains have been commonly detected in cats in Northeast China, with FBoV-1 being the more prevalent. FBoV-1 has been associated with outbreaks of hemorrhagic enteritis in domestic cats, providing direct evidence that this subtype can cause intestinal infection in cats. However, its pathogenic mechanisms remain unclear, and no commercially available vaccine is currently available to prevent FBoV infection. VP2 is the major capsid protein of FBoV and is an important immunodominant antigen. It contains multiple conserved antigenic epitopes and plays a key role in eliciting protective immune responses [22]. Moreover, VP2 is closely associated with the antigenicity and pathogenicity of bocaviruses and plays an important role in viral infection. Therefore, VP2 represents a promising target for the development of FBoV vaccines, with a strong theoretical basis and potential application value.

The BEVS is a highly efficient eukaryotic expression platform capable of producing high levels of recombinant proteins with appropriate post-translational modifications. The BEVS has been widely used for the production of recombinant proteins and subunit vaccines [23,24]. Among BEVS platforms, the Bac-to-Bac system is commonly used for heterologous protein production because of its advantages in protein folding and processing [25]. In this system, protein expression is driven by strong promoters, such as the polyhedrin (polh) and p10 promoters, enabling broad applications in the preparation of recombinant proteins, vaccines, and VLPs. Moreover, the Bac-to-Bac system can produce recombinant proteins at milligram-to-gram scales, making it suitable for industrial scale-up. In this study, the Bac-to-Bac system was used to express the FBoV VP2 protein, which can self-assemble into VLPs, and the immunogenicity of the resulting VLP vaccines was evaluated in mice.

In this study, three adjuvants, including Alum, ISA 206, and GEL 02, were selected to enhance the immunogenicity of the VP2 protein, and the resulting VP2 VLP vaccine formulations induced robust systemic immune responses in BALB/c mice. All immunized groups elicited VP2-specific IgG antibodies and neutralizing antibodies, indicating that VP2 VLPs possess favorable immunogenicity and can induce functional humoral immune responses. Among the tested formulations, VP2+GEL 02 induced the highest levels of VP2-specific IgG and neutralizing antibodies, suggesting that GEL 02 effectively enhanced the immunogenicity of VP2 VLPs. This finding is consistent with previous studies showing that appropriate adjuvants can substantially improve the immunogenicity of recombinant protein- or VLP-based vaccines. In particular, aqueous adjuvants have been reported to promote early and strong humoral immune responses, while VLP-based immunogens can efficiently induce both specific antibody responses and neutralizing activity [26,27]. In the present study, the IgG2a/IgG1 ratio remained below one, indicating a predominantly Th2-biased humoral immune response. In addition, immunization increased the proportions of mature and activated B cells, promoted splenic lymphocyte proliferation, and elevated the levels of immune-related cytokines. Flow cytometry further showed increased frequencies of CD4^+^ T cells, CD8^+^ T cells, and activated CD4^+^ CD69^+^ and CD8^+^ CD69^+^ T cells, particularly in the VP2+GEL 02 group. These results suggest that FBoV VP2 VLP vaccination not only induces potent antibody-mediated immunity but also activates lymphocytes and promotes cellular immune responses, which together may contribute to a broader antiviral immune profile.

In this study, the three adjuvant formulations exhibited distinct effects on the immune responses induced by VP2 VLPs. Among them, GEL 02 showed superior immunostimulatory activity, as demonstrated by enhanced VP2-specific antibody production, increased neutralizing antibody titers, stronger lymphocyte proliferation, and more pronounced GC responses. The improved performance of GEL 02 may be attributed to its ability to enhance antigen persistence, facilitate antigen uptake by antigen-presenting cells, and promote subsequent immune activation. Consistent with these findings, GEL 02-formulated VP2 VLPs exhibited prolonged antigen retention at the injection site and enhanced uptake by dendritic cells in our study, which may contribute to improved antigen presentation and stronger adaptive immune responses. Alum is widely used and effectively induces humoral immunity; its immune-stimulating capacity may be relatively limited compared with more advanced adjuvant systems, and it predominantly induces humoral and Th2-biased immune responses [28]. ISA 206, as an emulsion-based adjuvant, can prolong antigen exposure and enhance immune activation [20,29]. The Montanide GEL 02 adjuvant consists of highly stable sodium polyacrylate gel particles dispersed in water and has been shown to enhance vaccine-induced protection while maintaining a favorable safety profile [30,31]. These results suggest that the selection of appropriate adjuvants is critical for optimizing the immunogenicity of recombinant VLP-based vaccines.

The enhanced immune responses induced by the FBoV VP2 VLP vaccine may be partly explained by improved antigen retention, uptake, and presentation. *In vivo* fluorescence imaging showed that adjuvant-formulated VP2 VLPs persisted longer at the injection site than free VP2, suggesting the formation of an antigen depot. Such antigen retention may prolong antigen exposure, regulate antigen release, and facilitate the recruitment of antigen-presenting cells, thereby supporting sustained immune activation. Consistently, stronger fluorescence signals were detected in the spleens of mice receiving adjuvant-formulated VP2, especially in the VP2+GEL 02 group, suggesting enhanced antigen delivery to secondary lymphoid organs. Previous studies on nanoparticle and VLP vaccine platforms have shown that particulate antigens can improve antigen transport, lymphoid organ retention, and uptake by dendritic cells, thereby enhancing downstream adaptive immune responses [32,33]. Moreover, VP2 VLP formulations upregulated the expression of MHC-II and the costimulatory molecules CD80 and CD86 on BMDCs, indicating effective DC activation and maturation. Because mature DCs are essential for antigen presentation and T-cell priming, these findings suggest that the particulate structure of VP2 VLPs, together with adjuvant-mediated antigen retention and delivery, enhances antigen uptake and presentation by dendritic cells, thereby strengthening subsequent T- and B-cell responses.

The potent immunogenicity of the FBoV VP2 VLP vaccine was further supported by its ability to promote germinal center responses. GCs are central sites for T-cell-dependent humoral immunity, where activated B cells undergo proliferation, class-switch recombination, affinity maturation, and differentiation into plasma cells and memory B cells [34]. In this study, Ki67 immunohistochemical staining showed that immunization markedly increased proliferative signals in lymphoid tissues, indicating enhanced GC formation. Flow cytometry further demonstrated that VP2 VLP vaccination increased the proportions of GC B cells and Tfh cells, with the most pronounced responses observed in the VP2+GEL 02 group. Tfh cells provide essential help to GC B cells through costimulatory signals and cytokine-mediated regulation, thereby supporting B-cell proliferation, antibody affinity maturation, and the generation of long-lived humoral immunity [35]. Consistent with this mechanism, the VP2+GEL 02 group also showed increased proportions of plasma cells and memory B cells, suggesting enhanced B-cell differentiation and immunological memory formation. These findings indicate that the strong immune efficacy of the FBoV VP2 VLP vaccine may be associated with coordinated activation of the DC–T-cell–B-cell axis, particularly enhanced antigen presentation, Tfh-cell activation, GC B-cell responses, and GC formation.

This study has several limitations that should be acknowledged. First, the immunogenicity of the baculovirus-expressed FBoV VP2 VLP vaccine was evaluated in BALB/c mice rather than in cats, the natural host of FBoV. BALB/c mice were selected because they represent a well-established model for preliminary vaccine evaluation, with a well-characterized immune system and abundant immunological reagents that facilitate comprehensive analyses of vaccine-induced immune responses. Nevertheless, species-specific differences may limit the direct translation of these findings to cats. Second, although the vaccine elicited robust humoral and cellular immune responses in BALB/c mice, its protective efficacy was not evaluated by a viral challenge. The lack of an established FBoV challenge model, together with current laboratory constraints and the ethical requirements associated with challenge studies in cats, prevented direct assessment of vaccine-mediated protection. Therefore, the present study demonstrates the immunogenicity rather than the protective efficacy of the VP2 VLP vaccine. Third, virus neutralization assays were performed only against the homologous FBoV-1 strain ZZ202401, which represents the predominant circulating genotype. The cross-neutralizing activity of the vaccine against FBoV-2 and FBoV-3 was not investigated because representative isolates of these genotypes were unavailable in our laboratory. In addition, adjuvant-only control groups were not included, terminal tissue-based analyses were performed using three biological replicates per group, and only female BALB/c mice were used, which may have limited the comprehensive evaluation of vaccine-induced immune responses. Future studies will include adjuvant-only control groups to further clarify the independent contribution of each adjuvant to the observed immune responses. Despite these limitations, the present study provides the first systematic evaluation of the immunogenicity of a baculovirus-expressed FBoV VP2 VLP vaccine and offers a valuable foundation for subsequent vaccine development. Future studies will focus on evaluating the vaccine in cats, establishing an appropriate FBoV challenge model, assessing cross-genotype neutralizing activity, incorporating adjuvant-only control groups and both sexes in the experimental design, and further validating the immunogenicity, safety, and protective efficacy of the FBoV VP2 VLP vaccine.

## 5. Conclusions

In conclusion, this study successfully expressed and purified the FBoV VP2 protein using the baculovirus expression system, and the purified VP2 protein self-assembled into VLPs. These findings demonstrate that VP2 exhibits favorable immunogenicity and is a promising vaccine antigen for the development of FBoV vaccines. The FBoV VP2 VLP vaccine induced specific humoral and neutralizing antibody responses in mice, increased the proportions of CD4^+^ and CD8^+^ T lymphocytes, and promoted DC maturation. Moreover, the vaccine activated the Tfh–GC axis, thereby promoting the differentiation of GC B cells into plasma cells and memory B cells and effectively inducing immune memory. Given the current lack of a commercially available vaccine against FBoV, these findings provide new insights and technical support for the development of vaccines targeting FBoV infection, which may contribute to the prevention and control of FBoV-associated diseases.

## Data Availability

The data that support the findings of this study are available from the corresponding author upon request.

## References

[1] Hosie M.J., Hofmann-Lehmann R. (2022). Special Issue: Viral Infections in Companion Animals. Viruses.

[2] Ng T.F., Mesquita J.R., Nascimento M.S., Kondov N.O., Wong W., Reuter G., Knowles N.J., Vega E., Esona M.D., Deng X. (2014). Feline fecal virome reveals novel and prevalent enteric viruses. Vet. Microbiol..

[3] Zhang Q., Niu J., Yi S., Dong G., Yu D., Guo Y., Huang H., Hu G. (2019). Development and application of a multiplex PCR method for the simultaneous detection and differentiation of feline panleukopenia virus, feline bocavirus, and feline astrovirus. Arch. Virol..

[4] Stuetzer B., Hartmann K.J.V.J. (2014). Feline parvovirus infection and associated diseases. Vet. J..

[5] Yi S., Niu J., Wang H., Dong G., Zhao Y., Dong H., Guo Y., Wang K., Hu G. (2018). Detection and genetic characterization of feline bocavirus in Northeast China. Virol. J..

[6] Piewbang C., Kasantikul T., Pringproa K., Techangamsuwan S. (2019). Feline bocavirus-1 associated with outbreaks of hemorrhagic enteritis in household cats: Potential first evidence of a pathological role, viral tropism and natural genetic recombination. Sci. Rep..

[7] Brussel K.V., Wang X., Shi M., Carrai M., Li J., Martella V., Beatty J.A., Holmes E.C., Barrs V.R. (2020). Identification of Novel Astroviruses in the Gastrointestinal Tract of Domestic Cats. Viruses.

[8] Lau S.K.P., Woo P.C.Y., Yeung H.C., Teng J.L.L., Wu Y., Bai R., Fan R.Y.Y., Chan K.H., Yuen K.Y. (2012). Identification and characterization of bocaviruses in cats and dogs reveals a novel feline bocavirus and a novel genetic group of canine bocavirus. J. Gen. Virol..

[9] Yao X.Y., Shi B.W., Li H.P., Han Y.Q., Zhong K., Shao J.W., Wang Y.Y. (2024). Epidemiology and genotypic diversity of feline bocavirus identified from cats in Harbin, China. Virology.

[10] Manteufel J., Truyen U. (2008). Animal bocaviruses: A brief review. Intervirology.

[11] Liu C., Liu F., Li Z., Qu L., Liu D. (2018). First report of feline bocavirus associated with severe enteritis of cat in Northeast China, 2015. J. Vet. Med. Sci..

[12] Takano T., Takadate Y., Doki T., Hohdatsu T. (2016). Genetic characterization of feline bocavirus detected in cats in Japan. Arch. Virol..

[13] Zhang W., Li L., Deng X., Kapusinszky B., Pesavento P.A., Delwart E. (2014). Faecal virome of cats in an animal shelter. J. Gen. Virol..

[14] Piewbang C., Wardhani S.W., Phongroop K., Lohavicharn P., Sirivisoot S., Kasantikul T., Techangamsuwan S. (2022). Naturally acquired feline bocavirus type 1 and 3 infections in cats with neurologic deficits. Transbound. Emerg. Dis..

[15] Garigliany M., Gilliaux G., Jolly S., Casanova T., Bayrou C., Gommeren K., Fett T., Mauroy A., Lévy E., Cassart D. (2016). Feline panleukopenia virus in cerebral neurons of young and adult cats. BMC Vet. Res..

[16] Cotmore S.F., Agbandje-Mckenna M., Chiorini J.A., Mukha D.V., Pintel D.J., Qiu J., Soderlund-Venermo M., Tattersall P., Tijssen P., Gatherer D. (2014). The family Parvoviridae. Arch. Virol..

[17] Amimo J.O., Njuguna J., Machuka E., Okoth E., Djikeng A. (2017). First Complete Genome Sequences of Porcine Bocavirus Strains from East Africa. Genome Announc..

[18] Yang W.Z., Yu J.M., Li J.S., Cheng W.X., Huang C.P., Duan Z.J. (2012). Genome characterization of a novel porcine bocavirus. Arch. Virol..

[19] Ono C., Okamoto T., Abe T., Matsuura Y. (2018). Baculovirus as a Tool for Gene Delivery and Gene Therapy. Viruses.

[20] Zhang J., Wang P., Li Z., Xie Y., Jin N., Zhang H., Lu H., Han J. (2024). Adjuvant screening of the Senecavirus A inactivated vaccine in mice and evaluation of its immunogenicity in pigs. BMC Vet. Res..

[21] Wangkaghart E., Deville S., Wang B., Srisapoome P., Wang T., Secombes C.J. (2021). Immune response and protective efficacy of two new adjuvants, Montanide™ ISA 763B VG and Montanide™ GEL02, administered with a Streptococcus agalactiae ghost vaccine in *Nile tilapia* (*Oreochromis niloticus*). Fish Shellfish Immunol..

[22] Mietzsch M., Pénzes J.J., Agbandje-McKenna M. (2019). Twenty-Five Years of Structural Parvovirology. Viruses.

[23] Rong R., Li T., Zhang Y., Gu Y., Xia N., Li S. (2019). Progress in vaccine development based on baculovirus expression vector system. Sheng Wu Gong Cheng Xue Bao Chin. J. Biotechnol..

[24] Lin S.Y., Chung Y.C., Hu Y.C. (2014). Update on baculovirus as an expression and/or delivery vehicle for vaccine antigens. Expert Rev. Vaccines.

[25] Murphy C.I., Piwnicaworms H., Grünwald S., Romanow W.G., Francis N., Fan H.Y. (2004). Overview of the baculovirus expression system. Curr. Protoc. Mol. Biol..

[26] Khoury D.S., Cromer D., Reynaldi A., Schlub T.E., Wheatley A.K., Juno J.A., Subbarao K., Kent S.J., Triccas J.A., Davenport M.P. (2021). Neutralizing antibody levels are highly predictive of immune protection from symptomatic SARS-CoV-2 infection. Nat. Med..

[27] Zepeda-Cervantes J., Ramírez-Jarquín J.O., Vaca L. (2020). Interaction Between Virus-Like Particles (VLPs) and Pattern Recognition Receptors (PRRs) From Dendritic Cells (DCs): Toward Better Engineering of VLPs. Front. Immunol..

[28] Gołoś A., Lutyńska A. (2015). Aluminium-adjuvanted vaccines--a review of the current state of knowledge. Prz. Epidemiol..

[29] Gorse G.J., Grimes S., Buck H., Mulla H., White P., Hill H., May J., Frey S.E., Blackburn P. (2022). A phase 1 dose-sparing, randomized clinical trial of seasonal trivalent inactivated influenza vaccine combined with MAS-1, a novel water-in-oil adjuvant/delivery system. Vaccine.

[30] Xu Y., Wang Q., Wei B., Huang X., Wen Y., Yan Q., Ma X., Zhao Q., Cao S., Huang Y. (2019). Enhanced Immune Responses Against Japanese Encephalitis Virus Infection Using Japanese Encephalitis Live-Attenuated Virus Adjuvanted with Montanide GEL 01 ST in Mice. Vector Borne Zoonotic Dis..

[31] Lin Y., Ren G., Zhao J., Shao Y., He B., Tang X., Sha O., Zhao W., Liu Q., Xu L. (2022). Long-Term Protection Elicited by an Inactivated Vaccine Supplemented with a Water-Based Adjuvant against Infectious Hematopoietic Necrosis Virus in Rainbow Trout (*Oncorhynchus mykiss*). Microbiol. Spectr..

[32] Pulendran B., Arunachalam P.S., O’Hagan D.T. (2021). Emerging concepts in the science of vaccine adjuvants. Nat. Rev. Drug Discov..

[33] Oyewumi M.O., Kumar A., Cui Z. (2010). Nano-microparticles as immune adjuvants: Correlating particle sizes and the resultant immune responses. Expert Rev. Vaccines.

[34] Liu X., Liu B., Qi H. (2023). Germinal center reaction and output: Recent advances. Curr. Opin. Immunol..

[35] Shao W., Wang Y., Fang Q., Shi W., Qi H. (2024). Epigenetic recording of stimulation history reveals BLIMP1-BACH2 balance in determining memory B cell fate upon recall challenge. Nat. Immunol..

